# Supplementary material for: GWAS reveal a role for the central nervous system in regulating weight and weight change in response to exercise
Source: Sci Rep. 2021 Mar 4;11:5144. doi: 10.1038/s41598-021-84534-w (PMC7933348; doi:10.1038/s41598-021-84534-w)
Supplement: Supplementary file 1 — Supplementary Legends. [file 41598_2021_84534_MOESM1_ESM.docx]

Figure S1. There is no clear relationship between body weight and basal activity levels in the study population. Body weight per fly in mg (X-axis) is plotted against basal activity levels (Y-axis; from ^45^), with data from females shown in **A** and data from males in **B**. For both correlations, the p-value is not significant (p>0.05).

Figure S2. There is no clear relationship between body weight and lifespan in females of the study population. Body weight per fly in mg (X-axis) is plotted against lifespan (Y-axis). Lifespan data in **A** are from Durham and colleagues ^52^, while lifespan data in **B** are from Ivanov and colleagues ^53^. For both correlations, the p-value is not significant (p>0.05).

Figure S3. In females, the increase in overall activity associated with an exercise treatment is correlated to the weight change. The change in activity induced by a rotational exercise treatment over baseline (X-axis) is plotted against the change in body weight in mg (Y-axis). Data from females are shown in **A**, displaying a significant positive correlation (p = 0.007447), while data from males in **B** reveal no significant correlation (p = 0.3458).

Supplemental Table S1. Raw data used in this study. This file includes a list of all DGRP strains that are part of this study.

Supplemental Table S2. Quantitative genetics parameters. Various quantitative genetics parameters such as heritability are reported for weight in the control and treated animals.

Supplemental Table S3. GWAS results. This table includes the GWAS output for and information about genetic variants detected as significant in the GWASs.

Supplemental Table S4. GO term results. GO term results from the PANTHER database are reported for the genes identified in the GWAS for weight in control animals, in the GWAS for weight in the exercised animals, and in the GWAS for weight change with exercise treatment.

Supplemental Table S5. QMR data. This table includes the raw data from the QMR study presented in Figure 6.

Supplemental Table S6. Wing size data. This table includes the raw wing size data presented in Figure 7.
