# Supplementary figures and images for: GWAS reveal a role for the central nervous system in regulating weight and weight change in response to exercise
Source: Sci Rep. 2021 Mar 4;11:5144. doi: 10.1038/s41598-021-84534-w (PMC7933348; doi:10.1038/s41598-021-84534-w)

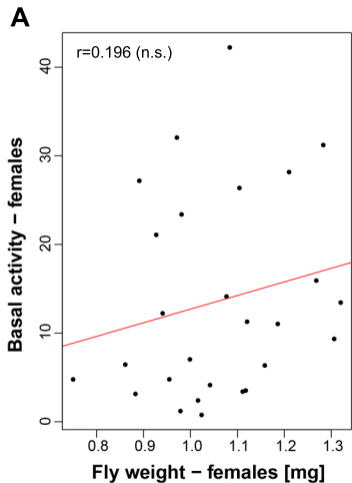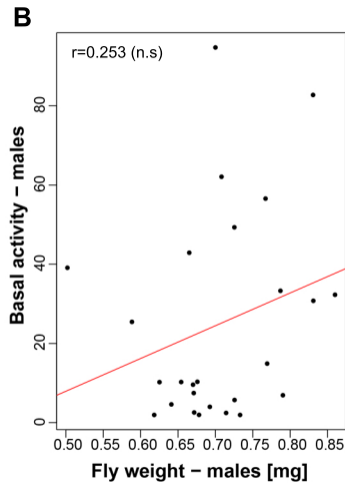

Supplement: Supplementary file 2 — Supplementary Figure 1. [file 41598_2021_84534_MOESM2_ESM.pdf]

Lifespan [days]

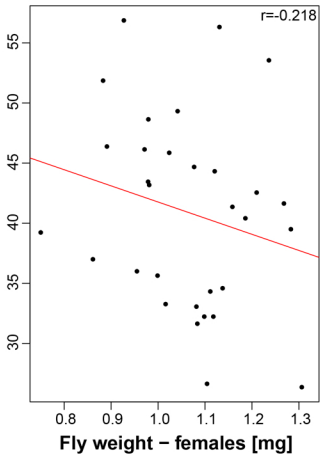

Lifespan [days]

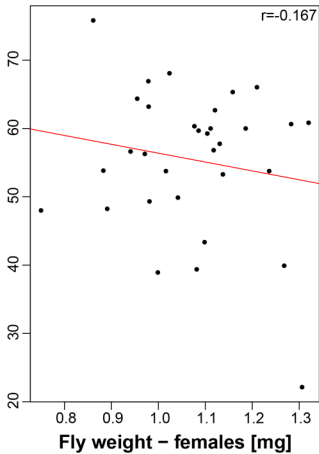

Supplement: Supplementary file 3 — Supplementary Figure 2. [file 41598_2021_84534_MOESM3_ESM.pdf]

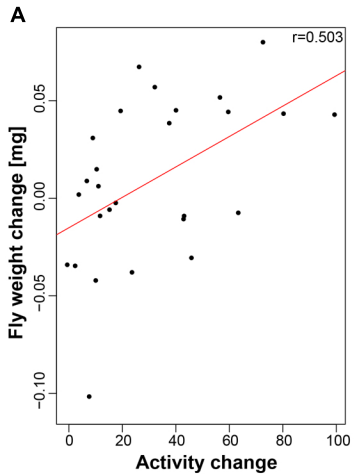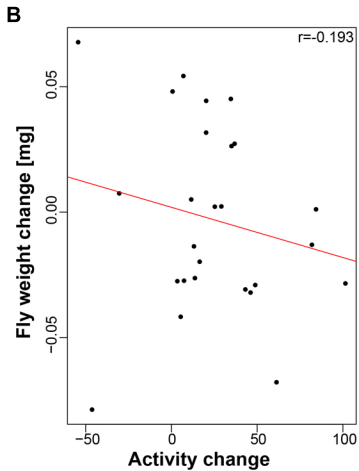

Supplement: Supplementary file 4 — Supplementary Figure 3. [file 41598_2021_84534_MOESM4_ESM.pdf]
